# Community-Acquired Escherichia coli Meningoencephalitis and Brain Abscess in an Immunocompromised Patient: A Case Report and Literature Review

**DOI:** 10.7759/cureus.87916

**Published:** 2025-07-14

**Authors:** Nikolaos Kalakos, Areti Kalfoutzou, Angeliki Katsarou, Theodora Zormpala, Maria K Papakonstantinou, Evangelia Ntaskagianni, Marina Kouveletsou, Spyros Sfikas, Foteini Protogerou, Adam Mylonakis, Panagiotis Petrikkos

**Affiliations:** 1 First Department of Internal Medicine, 251 Air Force General Hospital, Athens, GRC; 2 Department of Medical Oncology, 251 Air Force General Hospital, Athens, GRC; 3 Second Department of Internal Medicine, 251 Air Force General Hospital, Athens, GRC; 4 Department of Neurosurgery, 251 Air Force General Hospital, Athens, GRC; 5 Department of Neurology, 401 General Military Hospital of Athens, Athens, GRC; 6 First Department of Surgery, Laiko General Hospital, National and Kapodistrian University of Athens, Athens, GRC

**Keywords:** brain abscess, e. coli, immunocompromised patient, meningitis, meningoencephalitis

## Abstract

Gram-negative bacterial meningitis (GNBM) is rare, primarily affecting infants. *Escherichia coli* (*E. coli*) is the most common cause, with high mortality and poor clinical outcomes. We report a rare case of an immunocompromised 61-year-old male with a history of chronic lymphocytic leukemia who presented with altered mental status and fever. He was diagnosed with *E. coli *meningoencephalitis complicated by brain abscess, likely secondary to otitis media. Despite initial medical management, neurosurgical intervention was required due to abscess progression. The patient fully recovered following craniotomy, targeted antimicrobial therapy, and intravenous immunoglobulin administration. A comprehensive literature review was performed, including 142 cases of spontaneous community-acquired *E. coli *meningitis in adults from January 2000 to January 2024. The most common risk factors identified were urinary tract infection (24.4%), immunosuppression (22.1%), and diabetes mellitus (19.1%). Clinical manifestations frequently included fever (65.3%), altered mental status (50%), and neck stiffness (41.9%). Pneumocephalus was the only independent predictor of in-hospital mortality, with a 93% decrease in the odds of survival (odds ratio: 0.071; 95% CI: 0.005-0.942; p=0.045). Survivors had significantly lower CRP and higher cerebrospinal fluid (CSF) glucose levels compared to non-survivors. This case highlights the diagnostic and therapeutic challenges of community-acquired *E. coli *meningoencephalitis. Early recognition, appropriate antimicrobial treatment, and timely neurosurgical intervention are essential for optimal outcomes. Pneumocephalus, elevated CRP levels, and low CSF glucose may serve as predictors of poor prognosis.

## Introduction

Gram-negative bacterial meningitis (GNBM) is a rare etiology of central nervous system (CNS) infection usually encountered in infants [1-3]. The annual incidence of GNBM was estimated 2/100,000 adults in large prospective and retrospective studies [4]. Among the most common responsible bacteria for GNBM are *Escherichia coli* (*E. coli*) and *Klebsiella pneumoniae*, with sporadic cases from other Enterobacterales (non-typhoidal Salmonella*, *Serratia spp.*, Proteus mirabilis, Enterobacter cloacae*) and non-fermenters, such as Pseudomonas spp. and *Acinetobacter baumannii* [1-3]. *E. coli* is a rare cause of bacterial meningitis. Recent observational studies found that *E. coli *was among the most prevalent causes of GNBM in adult populations [3-6]. Moreover, the reported risk factors for *E. coli *meningitis are type 2 diabetes mellitus (T2DM), cancer, heart disease, liver and kidney dysfunction, myxedema, paralysis agitans, alcoholism, cirrhosis, *E. coli* septicemia, pulmonary tuberculosis (TB), otitis media, head trauma, recent abdominal surgery, dural defect surgery, and application of epidural catheter [7]. Also, older epidemiological studies reported mortality rates between 53% and 71.4% for patients with *E. coli* meningitis [7,8]. In a case series, 60% of the patients with *E. coli* meningitis experienced complications related to the disease, such as septic shock, disseminated intravascular coagulation, seizures, cerebral herniation, hydrocephalus, stroke, and hearing impairment [7]. *E. coli* in the cerebrospinal fluid (CSF) in patients with meningitis has been implicated as a prognostic factor of poor outcome [9]. So far, only one retrospective study is available for 45 cases of spontaneous community-acquired *E. coli* meningitis from 1946 to 2018 [10]. While Bichon et al. reported the number of cases, risk factors, treatment choices, and outcomes in *E. coli* meningitis, they provided little information on presenting symptoms or the clinical course, details that would help clinicians manage community-acquired *E. coli* meningitis in adults [10].

In this study, we report a rare case of community-acquired *E. coli *meningoencephalitis complicated by brain abscess in an immunocompromised adult associated with ear infection. Moreover, we present a comprehensive literature review of the observational studies (retrospective studies, prospective studies, case series, and case reports) of community-acquired *E. coli *meningitis from 2000 to January 2024. This review aimed to enlighten possible predisposing factors and clinical clues, as well as to underline the most effective treatment, and the potential complications associated with *E. coli*-induced CNS infection.

## Case presentation

A 61-year-old male patient, a resident of Greece, was admitted to the ED due to a decrease in the level of communication and expressive aphasia within the past two hours. The patient’s past medical history included chronic lymphocytic leukemia (CLL), treated with cyclophosphamide from June to September 2020, followed by rituximab from July 2020 to September 2021 (last dose was 24 months before presentation); hypertension; dyslipidemia; a surgically repaired left inguinal hernia; and a recent hospitalization due to herpetic cheilitis, treated with valaciclovir and prednisolone three months prior to ED admission.

At the ED, the patient had a blood pressure of 149/99 mmHg, a heart rate of 104 beats per minute, and a temperature of 39.1°C. The physical examination showed palpable submandibular lymph nodes, while no abnormal findings were revealed from the rest of the systems. During the examination, the patient presented generalized tonic-clonic seizures managed with diazepam and levetiracetam. The neurologic examination post-ictally revealed an abnormal Glasgow Coma Scale (GCS) score of 10/15 (E3V3M4), neck stiffness, and bilateral positive Brudzinski sign - findings suggestive of meningeal irritation - while other aspects of the neurologic examinations were limited due to altered consciousness. Papilledema was not observed by ophthalmologic examination. The laboratory analysis showed neutrophilic leukocytosis with a white blood cell (WBC) count of 14,000 cells/μL (normal range: 4.000-10.000 cells/μL) and neutrophils comprising 11,800 cells/μL, and an elevated C-reactive protein (CRP) (14.48, normal range: 0-10 mg/L) (Table 1).

**Table 1:** Patient’s blood differential and blood chemistry on admission.

Laboratory examination	Patient's results	Reference range
White blood cells	14,000 cells/μL	4,000-10,000 cells/μL
Neutrophil count	8,700 (62.4%) cells/μL	1,500-7,000 cells/μL
Hemoglobin	14.6 g/dL	14-18 g/dL
Hematocrit	44.1%	42-52%
Platelet count	151,000/μL	140,000-400,000/μL
C-reactive protein (CRP)	14.48 mg/L	0-10 mg/L
Blood urea nitrogen (BUN)	42 mg/L	15-54 mg/dL
Creatinine	0.9 mg/L	0.7-1.3 mg/dL
Sodium	142 mEq/L	137-150 mEq/L
Glucose	105 mg/L	75-110 mg/dL

The patient immediately received empiric antimicrobial treatment intravenously with ceftriaxone 2 g, ampicillin 2 g, vancomycin 1 g, and acyclovir 1 g, followed by maintenance dosing (ceftriaxone 2 g q12 h, ampicillin 2 g q4 h, vancomycin 1 g q12 h, acyclovir 750 mg q8 h). A computed tomography (CT) scan of the brain with intravenous contrast demonstrated hypodense imaging of the right frontal lobe surrounded by edema with subsequent obliteration of the subarachnoid spaces and total opacification of the right mastoid air cells, suggestive of right mastoiditis. A subsequent lumbar puncture (LP) and cerebrospinal fluid (CSF) analysis revealed an elevated cell count of 3,200 cells/μL, predominantly neutrophils (80%), a protein level of 309 mg/dL, and a glucose level of 47 mg/dL (Table 2). The clinical and laboratory results indicated bacterial meningitis. Because cerebral edema with raised intracranial pressure was suspected, the attending neurologist also ordered intravenous mannitol.

**Table 2:** Cerebrospinal fluid analysis of the patient upon admission.

Laboratory examination	Patients results	Reference range
Color	Colorless	Colorless
White blood cell count	3,200 cells/μL	0-4 cells/μL
Polynuclear	80%	-
Glucose	47 mg/dL	45-80 mg/dL
Total protein	309 mg/dL	15-45 mg/dL

The patient was hospitalized in the Department of Internal Medicine for CNS infection. The symptoms completely subsided within 24 hours of admission and antibiotic administration. Upon examination on the second day of admission, the patient was awake, mobile, and oriented, with a GCS score of 15/15 and transient amnesia for the past 12 hours. The multiplex molecular test of both CSF and blood samples was positive for *E. coli;* the CSF and blood cultures confirmed *E. coli* as the cause of the CNS infection, demonstrating full susceptibility to all antibiotics tested (appendix 1). The antimicrobial regimen was subsequently switched to ceftriaxone alone, based on the susceptibility test results.

In search of the underlying factors that caused GNBM, further examinations were performed. The additional performance of urine and stool cultures did not detect other pathogens. The fecal ova and parasite tests were negative. The quantitative determination of immunoglobulins revealed notable hypogammaglobulinemia (Table 3). Human immunodeficiency virus serology was also negative. An otorhinolaryngologist's estimation showed an injured right outer ear canal and median otitis; a nasal spray of corticosteroids was prescribed.

**Table 3:** Patient’s immunologic studies on admission.

Laboratory examination	Patient’s results	Reference range
IgA	30 mg/dL	85-385 mg/dL
IgG	222 mg/dL	564-1,770 mg/dL
IgG1	1.68 g/L	4.9-11.4 g/L
IgG2	0.51 g/L	1.5-4.6 g/L
IgG3	0.1 g/L	0.2-1.1 g/L
IgG4	0.09 g/L	0.08-1.4 g/L
Serum protein electrophoresis	Patient’s results	Reference range
Alpha 1	0.31 g/dL (5.54%)	0.1-0.4 g/dL (1.5-4.8%)
Alpha 2	0.64 g/dL (11.51%)	0.5-0.9 g/dL (8-14.8%)
Beta	0.51 g/dL (9.02%)	0.4-1.1 g/dL (7-14%)
Gamma	0.21 g/dL (3.79%)	0.5-1.6 g/dL (9-21%)
Serum protein immunofixation	Monoclonal IgG (κ) detected	-

On the third day of hospitalization, the patient underwent a contrast-enhanced magnetic resonance imaging (MRI) scan of the brain. The results indicated the presence of a well-defined focal lesion measuring 38 mm in the right temporal lobe, which displayed a smooth, thick ring of peripheral enhancement on post-contrast T1-weighted images and central diffusion restriction, accompanied by surrounding vasogenic edema, highly suggestive of a brain abscess. Afterward, according to the neurosurgeon's consultation, conservative management was proposed since the patient had already been afebrile and clinically stable. The antimicrobial treatment was modified to ceftriaxone 2 g BID and metronidazole 500 mg TID intravenously.

On the fifth day of admission, the patient complained of headache and nausea, along with a new episode of fever. An emergent CT scan of the brain with intravenous contrast demonstrated a significant enlargement of the abscess, now measuring 47 mm in size, along with compression on the right ventricle. The patient was referred to the neurosurgery department for craniotomy and intraparenchymal evacuation of the abscess. During the surgical procedure, approximately 25 mL of purulent material was aspirated. Subsequent cultures of the aspirate confirmed the presence of *E. coli* with the same susceptibility pattern. The post-operative course was uneventful, and the patient was transferred back to the internal medicine department after a brief stay in the intensive care unit (ICU).

After the surgery, the patient’s condition rapidly improved and remained stable without any symptoms or neurological sequelae throughout hospitalization. The patient received antimicrobial treatment (ceftriaxone and metronidazole) for three weeks post-surgery. A follow-up MRI scan of the brain with intravenous contrast demonstrated delayed healing of the abscess in the right temporal lobe with a maximum diameter of 25 mm.

Within the scope of the patient's immunosuppression due to hypogammaglobulinemia, the patient received 40 g intravenous immunoglobulin (IVIG) following the hematologist’s recommendation. A follow-up MRI scan of the brain with intravenous contrast six weeks after the surgery showed complete resolution of the brain abscess. The patient completed eight weeks of intravenous antibiotic treatment (ceftriaxone and metronidazole) and was discharged on oral moxifloxacin (400 mg SID) for four weeks, along with levetiracetam. A follow-up MRI scan of the brain was scheduled for four weeks post-discharge. During subsequent outpatient follow-up, a recurrence of CLL was identified, leading to his referral to the department of hematology for re-initiation of chemotherapy.

## Discussion

Literature review

Methodology

A search of Medline and the Cochrane Library was conducted for reviews, observational, and descriptive studies on adult cases with spontaneous community-acquired *E. coli* meningitis from January 2000 to January 2024. The keywords used were “*Escherichia coli*,” “*E. coli*,” “meningitis,” “meningoencephalitis,” “encephalitis,” “brain abscess,” “intracranial infection,” and “cerebral infection.” Only studies with positive CSF cultures for *E. coli* were included. Case reports or case series, as well as patients from observational studies with nosocomial *E. coli* meningitis, were excluded.

Statistical analysis of the results was performed using SPSS software version 26.0 (Armonk, NY: IBM Corp.). The level of statistical significance was set at 5% (p=0.05). Normality was checked using the Kolmogorov-Smirnov method, and the comparison of means for quantitative parameters that followed a normal distribution was carried out with the independent sample t-test. The comparison of means for quantitative parameters that did not follow a normal distribution was carried out with the Mann-Whitney U test (comparison of a quantitative variable in two categories of a qualitative variable). The correlation of a quantitative variable with a qualitative variable (two categories) was tested using one-way ANOVA. The correlation between qualitative variables was tested using Pearson’s chi-square test. Finally, for variables that showed a statistically significant correlation, linear regression models were provided to determine the relationship.

Results

A total of 193 cases with spontaneous community-acquired *E. coli *meningitis from 49 observational and descriptive studies were recorded, including six prospective studies, six retrospective studies, two case series, and 35 case reports (Table 4) [4,9-23,10,21,22,23-56]. Studies were excluded if they involved cases of nosocomial or post-traumatic *E. coli *meningitis. Additionally, studies reporting negative CSF cultures for *E. coli*, cases describing *E. coli*-associated cerebral abscess or subdural empyema without confirmed meningitis, and reports of mixed bacterial meningitis (involving *E. coli *and other pathogens) were also not included in this review [57-65]. The clinical outcome of the cases was documented in 42 studies, encompassing 138 cases. Of these, 53 patients died, representing 38.4% of the cases reported in Table 4.

**Table 4:** Reported number of patients with spontaneous community-acquired E. coli meningitis from 2000 to January 2024. Year, location, type of studies, and clinical outcome of the cases included in the review.

Reference	Year	Country	Study type	Number of cases	Survived (n)
Bodilsen et al. [11]	2018	Netherlands	Prospective	36	23
van Veen et al. [14]	2016	Netherlands	Prospective	1	Not determined
Pomar et al. [4]	2013	Spain	Prospective	15	Not determined
Dzupova et al. [9]	2009	Czech Republic	Prospective	8	Not determined
Cabellos et al. [12]	2009	Spain	Prospective	6	1
van de Beek et al. [13]	2004	Netherlands	Prospective	4	Not determined
Hsiao et al. [17]	2021	Taiwan	Retrospective	12	7
Bouadma et al. [15]	2006	France	Retrospective	21	Not determined
Yang et al. [20]	2005	Taiwan	Retrospective	6	4
Chen et al. [16]	2005	Taiwan	Retrospective	8	6
Rau et al. [19]	2002	Taiwan	Retrospective	3	2
Huang et al. [18]	2002	Taiwan	Retrospective	4	Not determined
Moussiegt et al. [22]	2022	France	Case series	29	14
Lu and Chang [21]	2000	Taiwan	Case series	4	3
Komsani et al. [45]	2024	USA	Case report	1	1
Mir et al. [48]	2023	USA	Case report	1	1
Ray et al. [51]	2023	India	Case report	1	1
Memia et al. [46]	2023	USA	Case report	1	1
Amulele et al. [26]	2023	Kenya	Case report	1	1
Present case	2023	Greece	Case report	1	1
Jeter et al. [37]	2022	USA	Case report	1	1
Hurn et al. [35]	2021	UK	Case report	1	1
Hare et al. [34]	2021	Ireland	Case report	1	1
Dhir et al. [30]	2020	India	Case report	1	-
Zafar et al. [56]	2020	Pakistan	Case report	1	1
Vale et al. [54]	2019	Portugal	Case report	1	1
Bichon et al. [10]	2018	France	Case report	2	1
Algaba et al. [27]	2019	Spain	Case report	1	1
Kasimahanti et al. [40]	2018	India	Case report	1	1
Khanna et al. [42]	2018	USA	Case report	1	1
Tosa et al. [53]	2018	Japan	Case report	1	1
Dahal et al. [29]	2017	USA	Case report	1	-
Ishida et al. [36]	2016	Japan	Case report	1	1
Kohlmann et al. [44]	2015	Germany	Case report	1	1
Kobayashi et al. [43]	2013	Japan	Case report	1	Not determined
Kenzaka and Ueda [41]	2013	Japan	Case report	1	1
Abhilash and Gigin [23]	2013	India	Case report	1	1
Kangath and Midturi [39]	2013	USA	Case report	1	1
Elaldi et al. [31]	2013	Turkey	Case report	1	1
Gon et al. [33]	2012	Japan	Case report	1	1
Weyrich et al. [55]	2012	France	Case report	1	-
Lasboo et al. [25]	2010	USA	Case report	1	-
Miletic et al. [47]	2008	Croatia	Case report	1	1
Adamides et al. [24]	2007	Australia	Case report	1	-
Sule and Tai [52]	2007	Singapore	Case report	1	1
Chang et al. [28]	2006	Taiwan	Case report	1	-
Fujitani et al. [32]	2005	-	Case report	1	-
Passeron et al. [50]	2004	France	Case report	1	1
Johnson et al. [38]	2003	USA	Case report	1	-
Mofredj et al. [49]	2000	France	Case report	1	1

The median age, gender, underlying risk factors, and clinical presentation of the patients with community-acquired meningitis included in this review are shown in appendix 2. The median age of 87 patients with spontaneous community-acquired *E. coli *meningitis, reported across three observational studies and two case series, ranges from 57 to 69 years (age range: 33-89 years) [11,17,20-22]. The median age of 35 cases is 63 years old (age range: 34-91 years) in 33 case reports, and the only patient with spontaneous *E. coli *meningitis in a prospective study [10,14,23-25,27-31,33-50,52-56]. Seventy-one males (58%) and 51 females (42%) were reported in 39 epidemiological and descriptive studies (total 122 cases).

The medical histories and possible risk factors (including an active distant site of infection) were recorded in 43 studies, encompassing 131 cases of spontaneous, community-acquired *E. coli *meningitis, and are summarized in Table 5.

**Table 5:** Medical history and risk factors of community-acquired E. coli meningitis cases from January 2000 to January 2024. UTI: urinary tract infection; COVID-19: coronavirus disease-2019; HIV: human immunodeficiency virus; CNS: central nervous system; CSF: cerebrospinal fluid; COPD: chronic obstructive pulmonary disease; CKD: chronic kidney disease

Medical history and risk factors	n (%)	References
Active distant infections		
UTI by *E. coli*	32 (24.4)	[11,22-24,27,36,38,39,41,43,46]
UTI by other organism	14 (10.7)	[11,20]
Gastroenteritis (4/5 with strongyloidiasis)	5 (3.8)	[22,29,48]
Ear infection	4 (3.1)	[11,31,53] and present case
Pneumonia	3 (2.3)	[11]
Chronic otitis	2 (1.5)	[10,31]
Septic arthritis	2 (1.5)	[11]
Endocarditis	2 (1.5)	[21,41]
Spondylodiscitis	1 (0.8)	[25]
Psoas abscess	1 (0.8)	[11]
Aspergillus sphenoid sinusitis	1 (0.8)	[50]
COVID-19	1 (0.8)	[35]
Chronic cholecystitis	1 (0.8)	[52]
Immunosupression		
Immunosuppresive drugs	14 (10.7)	[10,11,29,34,45,54]
Hematological cancer	7 (5.3)	[17,20,21]
Solid cancer	6 (4.6)	[11,17]
HIV infection	1 (0.8)	[11]
Kidney transplantation	1 (0.8)	[14]
Undetermined	6 (4.6)	[22]
Metabolic disorders		
Diabetes mellitus	25 (19.1)	[11,17-24,29,33,46,48]
Hyperlipidemia	2 (1.5)	[46] and present case
Obesity	1 (0.8)	[44]
Cardiovascular comorbidities		
Hypertension	9 (6.9)	[17,23,35,41,46,48,52,54]
Cerebral infarct	6 (4.6)	[17,20,21]
Old cerebral infarcts	6 (4.6)	[17,20,21]
Atrial fibrillation	2 (1.5)	[45,53]
Cardiomyopathy	1 (0.8)	[45]
Structural CNS abnormalities		
CSF leakage in the past	2 (1.5)	[11,40]
Vertebral disk herniation	1 (0.8)	[44]
Spinal stenosis	1 (0.8)	[43]
Anterior sacral meningocele	1 (0.8)	[47]
Other disorders		
Alcohol abuse	21 (16)	[10,11,17,18,20-23,26,38,46,49,50,55]
Smoking history	6 (4.6)	[23,26,37,53,55]
Liver disease	4 (3.1)	[11,23,37]
Liver cirrhosis	2 (1.5)	[17,38]
Cholesteatoma	1 (0.8)	[53]
COPD	1 (0.8)	[44]
Pancreatitis	1 (0.8)	[11]
Cholelithiasis	1 (0.8)	[37]
Multiple sclerosis	1 (0.8)	[17]
Ankylosing arthritis	1 (0.8)	[34]
Rheumatoid arthritis	1 (0.8)	[29]
Gout	1 (0.8)	[45]
Lupus erythematous	1 (0.8)	[54]
Substance abuse	1 (0.8)	[17]
CKD on hemodialysis	1 (0.8)	[20]
Non-dialysis dependent CKD	1 (0.8)	[45]
Multiple UTIs and nephrolithiasis	1 (0.8)	[45]
Marfan syndrome	1 (0.8)	[39]
Anemia of chronic disease	1 (0.8)	[45]

Upon admission, the median GCS score for 20 cases with spontaneous community-acquired *E. coli* meningitis was 11 (range: 6-15), according to 19 case reports [10,24,30,36,37,39-42,45,46,48-50,53-56]. The median GCS scores in an observational study and a case series are 13 and 12, respectively [11,22]. Moreover, 12 patients (19.1%, n=63) had GCS score of eight or less upon admission [10,11,23-25,28,30,33,35-42,45,46,48-50,52-56] and present case (appendix 2). Additional clinical manifestations observed at admission are summarized in Table 6 and the present case [10,11,14,17,20-50,52-56].

**Table 6:** Presenting clinical manifestations. N: the total number of evaluable patients for each specific symptom

Manifestation	n/N with data	% of evaluable patients
Fever	81/124	65.3
Altered mental status	62/124	50.0
Neck stiffness	52/124	41.9
Headache	41/124	33.1
Nausea/vomiting	35/123	28.5
Seizures	14/124	11.3
Classic triad (fever, neck stiffness, headache)	4/35	11.4

Laboratory studies on admission were reported in 37 studies, including 115 cases of spontaneous community-acquired *E. coli *meningitis (appendix 3). The median values for CRP, WBC count, CSF leukocyte count, CSF protein and glucose levels, and the culture results are shown in Table 7.

**Table 7:** Laboratory investigation in patients with spontaneous, community acquired E. coli meningitis from January 2000 to January 2024. CRP: C-reactive protein; WBC: white blood cells; CSF: cerebrospinal fluid

Laboratory studies, study type, and references	Value/results, n
Median CRP (mg/dL) 1 prospective study [11]	230 (IQR: 80-321, n=36)
Median CRP (mg/dL) 1 case series [22]	240 (IQR: 89-356, n=29)
Median CRP (mg/dL) 11 case reports [10,24,31,33,35,36,44,52-55] and present case	149.5 (IQR: 71-306, n=12)
Median WBC (count/µL) 1 prospective study [11]	12,600 (IQR: 6,300-20,900, n=36)
Median WBC (count/µL) 1 case series [22]	7,600 (IQR: 6,400-12,900, n=29)
Median WBC (count/µL) 25 case reports [10,23-25,29-31,33,35,37,40,42,44-46,48-50,52-56,65]	13,700 (IQR: 11,600-19,150, n=25)
Median CSF leukocyte (count/mm^3^) and type 1 prospective study [11]	2,527 (IQR: 125-7,773, n=30)
Median CSF leukocyte (count/mm^3^) and type 2 case series [21,22]	5,310 (IQR: 1,355-12,960, n=4) [21], 2,340 (IQR: 520-5100), 90% neutrophils (n=29) [22]
Median CSF leukocyte (count/mm^3^) and type 25 case reports [10,23,25,28,29,31,33,36-42,44-46,48-50,52-56], 1 prospective study [14], and present case	1,055 (IQR: 287-4,257), 85% neutrophils (n=30)
Median CSF protein level (mg/dL) 1 prospective study [11]	340 (IQR: 230-600, n=33)
Median CSF protein level (mg/dL) 2 case series [21,22]	313.5 (IQR: 122.5-627, n=4) [21], 360 (IQR: 250-820, n=29) [22]
Median CSF protein level (mg/dL) 22 case reports [10,14,26-29,31,33,36,37,39,41,42,45,46,48-50,52-56]	309 (IQR: 114-423, n=24)
Median CSF glucose level (mg/dL) 2 case series [21,22]	56 (IQR: 30.5-93, n=4) [21], 12.6 (IQR: 1.9-36, n=29) [22]
Median CSF glucose level (mg/dL) 22 case reports [10,14,26-29,31,33,36,37,39,41,42,45,46,48-50,52-56]	37 (IQR: 5.04-55.8, n=19)
Blood cultures positive for *E. coli* [10,11,14,17,21-31,33,35-46,48-50,52-56]	64.9% (n=74)
Urine cultures positive for *E. coli* [10,11,14,17,21-31,33,35-46,48-50,52-56]	26.6% (n=28)
Ear discharge culture positive for *E. coli* [31,53]	n=2
Stool cultures positive for *E. coli* [31,53]	n=2
Stool cultures positive for Strongyloides [22,29]	n=4
Abscess collection positive for *E. coli* [44] and present case	n=2
Subdural collection positive for *E. coli* [24]	n=1
Pus from intervertebral disc collection positive for *E. coli* [25]	n=1
Ventricular collection positive for *E. coli* [34,43]	n=2

Imaging studies on admission or during hospitalization were recorded in 36 studies as described in appendix 3. Brain imaging was performed in 62 cases (83.8%) (brain CT was performed in 60 patients, brain MRI was performed in two cases) with spontaneous, community-acquired *E. coli* meningitis. Abnormalities in brain imaging were revealed in 26 cases (41.9%) with meningitis. Findings in other imaging studies are as follows: (a) discitis or spondylitis in four patients [25,33,41,44], (b) arachnoiditis in a patient [35], (c) pyelonephritis in three cases [23,33,46], (d) renal subcapsular hematoma in a case [45], (e) nephrolithiasis in two patients [40,45], (f) non-CNS abscess in three cases [25,37,44], (g) osteomyelitis or arthritis in two patients [38,54], (h) colitis in a patient [29], (i) meningoceles in a case [39], (j) mycotic aneurysm in a patient [55], (k) non-CNS infarcts in two cases [25,55], (l) sphenoidal sinusitis in a patient [50], (m) sacroiliitis on a patient [54], and (n) endocarditis in a case [41].

The complications, presence of concomitant distant infection foci, causes of death, and the treatment choices for patients with community-acquired *E. coli *meningitis are described in appendix 4. During hospitalization, complications were reported in 71 patients (56%) with spontaneous community-acquired *E. coli *meningitis in 43 studies (n=127) as summarized in Table 8.

**Table 8:** In-hospital complications among 127 adult cases with spontaneous, community-acquired E. coli meningitis.

Complication	n (%)	References
Any organ failure	10 (7.9)	[22,38]
New cerebral infarction	10 (7.9)	[11,41,52]
Brain abscess	8 (6.3)	[17,19-21,27,37] and present case
Hydrocephalus	6 (4.7)	[11,17,31]
Ventriculitis	5 (3.9)	[33,34,40,41,43]
Septic shock	5 (3.9)	[21,27,36,55]
Cerebritis	4 (3.1)	[11,27] and present case
Pneumocephalus	4 (3.1)	[24,25,30,41]
Hyponatraemia	4 (3.1)	[17,37,48]
Unspecified neurological complication	4 (3.1)	[22]
Mastoiditis	3 (2.4)	[31,53] and present case
Hyperosmolar hyperglycemic state	3 (2.4)	[20,21,41]
Disseminated intravascular coagulation	2 (1.6)	[33,36]
Nosocomial pneumonia	2 (1.6)	[35,44]
Osteomyelitis	2 (1.6)	[38,54]
Generalised brain edema	2 (1.6)	[11]
Subdural empyema	1 (0.8)	[24]
Cerebrospinal-fluid leakage	1 (0.8)	[31]

Among the survivors, neurological sequelae and other complications at discharge were recorded in 13 patients (16.7%), comprising slight to severe neurological disability in eight cases (10.3%) [11,17], hearing impairment in two patients (2.6%) [14,35], anosognosia in a patient (1.3%) [10], hemiparesis in a case (1.3%) [21], dysphagia in a patient (1.3%) [21], and lower limb motor impairment in a case (1.3%) [35].

The concomitant distant focus of infection was investigated in 102 patients with spontaneous community-acquired *E. coli* meningitis (35 studies). Concomitant UTI was reported in 37 patients (36.3%, 21 cases with UTI by *E. coli *and 16 cases with UTI of non-defined/reported pathogen) [10,11,22-25,27,33,36,38,39,41,43,45,46,50], ear infection was reported in six patients (4.9%, three cases with chronic otitis, a case with undefined ear infection, and two cases with acute media otitis) [10,11,21,31,53], and present case.

Among eight patients with *E. coli *meningitis who died, the mean±SD time to death was 21.1±16.9 days [10,20,25,28,29,38,55]. Deaths due to systematic complications were reported in 18 patients across eight studies and included sepsis in 10 patients (55.5%) [10,11,25,30], pneumonia in three cases (16.7%) [11], respiratory failure in two patients (11.1%) [11], hemorrhagic and hypovolemic shock in a patient each [28,55].

The initial empiric medical treatment is reported in 29 studies (54 patients) (Table 9). The suitable medication was reported for 60 patients across 31 studies. Third-generation cephalosporins remained the most common choice prescribed in 44/60 cases (73.3%; 12 cases as monotherapy) [10,14,22,24,26-29,34,36,37,39,40,42,43,45,46,48,52,54] and the present case. Carbapenems were the next most frequent class (9/60, 15%; two cases as monotherapy) [23,30,31,33,36,44,53,55,56], followed by aminoglycosides [30,31,33,53] and penicillins (including one course of amoxicillin) each used in 4/60 patients (6.7%) [27,28,49,50]. Fluoroquinolones [46,50,55] and metronidazole were each employed in three patients (5%) [24,37]. Fifty-five patients (57.3%) received appropriate initial medical treatment [10,11,14,18,20,21,23,24,27-31,33-36,38,40,42-56] and present case 25 studies (94 patients) report data about the antimicrobial susceptibility of *E. coli *[10,11,20,22-24,26-29,31,33,36,39,40,42,44,45,48,52-56] and present case. Seven cases (7.4%) with extended-spectrum beta-lactamase (ESBL) producing *E. coli* were recorded [22,31,33,44,53,55]. The susceptibility rates to ampicillin, ceftazidime, ceftriaxone, cefepime, meropenem, and ciprofloxacin were 10.8% (10/93), 8.6% (8/93), 10.8% (10/93), 7.5 % (7/93), 1.6% (1/64), and 5.9% (5/85), respectively [10,11,20,22-24,26-29,31,33,36,39,40,42,44,45,48,52-56].

**Table 9:** Initial empiric antimicrobial and antiviral therapy in 56 adult cases with spontaneous, community-acquired E. coli meningitis.

Empiric therapy	Patients, n (%)	References
3rd/4th-generation cephalosporin	49 (90.7)	[10,14,17,27-29,31,33,35,40,42-46,48-50,52-56] and present case
Vancomycin	31 (57.4)	[17,30,42,48,53,56] and present case
Penicillin derivatives (amoxicillin/penicillin/ampicillin/piperacillin-tazobactam)	18 (33.3)	[10,14,27-29,35-38,40,44,45,48,53-55] and present case
Acyclovir	7 (13.0)	[10,40,45,48,54]
Aminoglycoside	2 (3.7)	[30,55]
Meropenem	2 (3.7)	[23,33]
Metronidazole	1 (1.9)	[46]

Among those who survived (26 patients in case reports with one patient each) and those who died, the mean±SD duration of treatment was 29.4±13.7 days and 20.6±11.6 days, respectively [10,23-31,35-40,44-46,48-50,52-55] and present case. Surgical treatment is reported in eight cases [24,31,34,43,44,47,50] and present case. ICU admission and the use of mechanical ventilation were recorded in 52 patients (49%) and 16 patients (20.1%), respectively, as reported in 39 studies [10,11,14,18,22-31,33-56] and present case. Finally, anti-edematous therapy was used in three cases [42,53,55] and present case, while corticosteroid treatment was reported in 57 patients [11,22,42,53].

Among 37 cases of spontaneous community-acquired *E. coli* meningitis, statistical analysis showed significantly lower CRP levels (mean±SD: 142.78±133.56 mg/L) and higher CSF glucose levels (40.56±32.32 mg/dL) in survivors compared to non-survivors (CRP: 314±19.69 mg/L; CSF glucose: 2.61±3.43 mg/dL). These differences were statistically significant, with t=8.926, p=0.005 for CRP, and t=-4.624, p<0.001 for CSF glucose and the present case (Table 10) [10,14,23-42,44-56]. Moreover, appendix 5 and 6 show the correlation of gender, risk factors, clinical manifestations, CT findings, complications, treatment choices, and antibiotic resistance with survival. The presence of pneumocephalus was moderately associated with lower survival rate (r=-0.436, t=-2.657, χ² {degree of freedom: 1, the sample size: N=32}=6.095, p=0.014) and present case (appendix 6) [10,14,23-42,44-56]. Logistic regression analysis further confirmed that pneumocephalus was the only statistically significant predictor of in-hospital mortality, with a 93% decrease in the odds of survival (OR=0.071, 95% CI=0.005-0.942, Wald statistic=4.021, p=0.045) (appendix 7). Sensitivity analysis adjusting for age and gender preserved the significance of pneumocephalus as a predictor (p=0.047).

**Table 10:** Results of mean±SD, t- and p-values for age, GCS, vital signs, and laboratory investigations between survivors and non-survivors with E. coli meningitis. SBP: systolic blood pressure; WBC: white blood cells; CRP: C-reactive protein; CSF: cerebrospinal fluid; GCS: Glasgow Coma Scale

Variables	Survivors (mean±SD)	Non-survivors (mean±SD)	t-Value	p-Value
Age (years)	60.73±13.78 (N=26)	63.38±20.66 (N=8)	0.339	0.677
Initial GCS	10.94±3.087 (N=16)	11±2.7 (N=4)	0.040	0.971
Admission SBP (mmHg)	132.76±22.71 (N=17)	120.67±19 (N=3)	-0.985	0.398
Admission heart rate (beats/min)	100.38±23.58 (N=16)	150 (N=1)	2.041	0.059
WBC (k/µL)	14938.33±6538.54 (N=18)	15578.57±9658.49 (N=7)	0.162	0.849
CRP (mg/L)	142.78±133.56 (N=9)	314±19.69 (N=3)	8.926	0.005
CSF glucose levels (mg/dL)	40.56±32.32 (N=17)	2.61±3.43 (N=2)	-4.624	0.000

*E. coli *is a well-established pathogen responsible for GNBM in neonates and infants [2]. Even though there is no significant difference in the global number of *E. coli *cases or deaths caused by *E. coli *between 1990 and 2019 in adults [66], *E. coli* meningoencephalitis remains a serious and fatal infection, with mortality rates reaching up to 70% [7,8]. Interestingly, in our study among 138 patients with spontaneous community-acquired *E. coli *meningoencephalitis, the mortality rate was 38.4%, which is lower than the relevant rate reported in the previous literature.

Predisposing factors for GNBM are patient’s advanced age, the presence of diabetes mellitus (DM), alcoholism, cirrhosis, chronic urinary tract infection (UTI), otitis, mastoiditis, sinusitis, head trauma with CSF rhinorrhea or otorrhea, chronic renal disease, history of cancer, infection acquired in hospital, myeloma, leukemia and post-surgical situations (craniotomy, ventricular drainage, endovascular coil embolization) [4,5,67-70]. In this review with the 141 patients, the most common potential risk factors included concomitant UTI caused by *E. coli *(24.4%) [11,22-24,27,36,38,39,41,43,46] or other pathogen (10.7%) [11,20], immunosuppression (22.1%), DM (19.1%) [11,17-24,29,33,46,48], alcohol abuse (16%) [10,11,17,18,20-23,26,38,46,49,50,55], hypertension (6.9%) [17,23,35,41,46,48,52,54], older cerebral infarct (4.6%) [17,20,21], smoking history (4.6%) [23,26,37,53,55], concomitant gastroenteritis (3.8%), and concomitant ear infection (3.1%) [11,31,53]. However, none of these factors were associated with increased mortality. Our patient was immunocompromised based on his past medical history of CLL and low immunoglobulin levels, and he was proven to have concomitant ear infection.

*E. coli* infects the meninges through various mechanisms. *E. coli *can survive in extracellular environments and has adhesive abilities that enable it to interact with brain endothelial cells and eventually penetrate CNS barrier [71]. Such invasion usually requires a prolonged or high-grade bacteraemia [71]. Neonates are characterized by an immature innate immune system, which likely allows for higher levels of bacteriemia and an increased risk of *E. coli *meningitis [71,72]. A similar situation arises in immunocompromised adults (those receiving corticosteroids, cytotoxic chemotherapy, or those with hematological malignancies) because neutrophil dysfunction and complement depletion slow bacterial clearance and extend the duration of bacteremia [71]. This impaired defence probably underlies the higher incidence of *E. coli *meningitis observed in these populations [71]. Lastly, there are three potential mechanisms for bacterial abscess development: (1) direct spread from a local infection site (usually in cases of otitis media, mastoiditis, sinusitis, and dental infection), (2) as a complication of head trauma or surgery, and (3) after hematogenous spread from an infection elsewhere in the body [73].

The incidence of bacterial brain abscess is 0.3-1.3/100,000 person per year [73]. Recognized risk factors include otitis media and mastoiditis (associated with 30% of brain abscesses), sinusitis, chest pyogenic infections, head trauma, neurosurgical and dental procedures, and infections [73]. The most common pathogens in immunocompetent patients are Streptococcus spp., anaerobes, and staphylococci, while Enterobacteriaceae are responsible for 25% of brain abscesses [73]. Despite the administration of targeted antimicrobial treatment, the mortality rate is less than 15% and serious complications such as seizures, weakness, aphasia, and cognitive impairment may persist in more than 20% of cured patients [73].

In patients with otitis, bacterial pathogens are usually involved secondarily as a superinfection following a viral infection [74]. Common complications of otitis media include mastoiditis, labyrinthitis, facial nerve palsy, sigmoid sinus thrombosis, meningitis, and brain abscess [74]. Among 2,346 patients with otitis media, brain abscess was the most common and severe intracranial complication, occurring in 1.8% of the patients, while meningitis was found in 0.7% of the individuals [75]. Previous case series of bacterial meningitis associated with acute otitis did not isolate *E. coli* [76]. Since 2000, there have been four reported cases of spontaneous community-acquired *E. coli *meningitis in patients with otitis [10,11,31,53]. The underlying risk factors in these patients were alcohol abuse (n=1) [10], corticosteroid therapy (n=1) [10], and the presence of cholesteatoma (n=1) [53]. This is the first case of *E. coli *meningitis and brain abscess in an immunocompromised patient with otitis media.

The clinical presentation of *E. coli *meningoencephalitis varies widely, ranging from completely asymptomatic to a potentially life-threatening condition characterized by septic shock or coma. In this review the most common manifestations are fever (65.3%), altered mental status (50%), neck stiffness (41.9%), headache (33.1%), and nausea/vomiting (28.5%), while the classic meningitis triad was recorded in 11.4% of the patients similarly to an older prospective study [4]. In contrast, the clinical triad is found in >80% of patients with bacterial meningitis from more common pathogens [68]. Finally, headache and septic shock were not associated with poorer outcome compared to older studies [17,22]. Our patient presented to the ED with the three most common symptoms (altered mental status, fever, and neck stiffness).

Laboratory examinations are usually suggestive of a bacterial infection. Common findings include a high leukocyte count with a predominance of polymorphonuclear cells, along with elevated CRP and erythrocyte sedimentation rate (ESR) [77,78]. In this review, the median WBC and CRP values in 25 cases of *E. coli* meningitis were 13,700/µL (IQR: 11,600-19,150) and 149.5 mg/dL (IQR: 71-306), respectively [10,23-25,29-31,33,35,37,40,42,44-46,48-50,52-56,65]. Older observational studies reported median WBC counts and CRP values of 12,600 cells/µL (IQR: 6,300-20,900), 7,600 cells/µL (IQR: 6,400-12,900) and 230 mg/dL (IQR: 80-321), 240 mg/dL (IQR: 89-356), respectively [11,22]. Moreover, we found a significant difference in mean CRP value between survivors and non-survivors in 12 cases of *E. coli* meningitis [10,14,23-42,44-56]. CSF examination usually reveals polymorphonuclear leukocytosis, low glucose levels, and elevated protein concentration [21,22]. In our study of 30 cases of *E. coli *meningitis, the median CSF WBC count, glucose, and protein levels were 1055 cells/mm³ (85% neutrophils), 37 mg/dL (IQR: 5.04-55.8), and 309 g/dL (IQR: 114-423), respectively [10,23,25,28,29,31,33,36-42,44-46,48-50,52-56]. Previous prospective studies and case series report median CSF WBC counts, glucose, and protein levels ranging from 2,527 cells/mm^3^ to 5,310 cells/mm^3^, 12.6-56 mg/dL, and 313.5-360 mg/dL, respectively [11,21,22]. Survivors compared to non-survivors had significantly higher mean CSF glucose levels. This may be associated with a higher glycolytic rate by WBCs and the bacteria in CSF in patients with more severe inflammation [79]. The causative bacterium in brain abscesses can be detected in blood cultures in up to 28% of cases [77]. Definitive diagnosis of meningitis relies on the identification of *E. coli *via culture or PCR analysis on a CSF sample [68]. In cases of brain abscesses, intravenous antibiotics may be sufficient, but stereotactic aspiration (ideally performed before therapy to obtain microbiological samples) is preferred for small (<2 cm), deep-seated, or multiple lesions, whereas craniotomy with complete excision is reserved for large, solitary, readily accessible abscesses [78,80]. In this review, *E. coli *was isolated from the blood of 64.9% of the patients, followed by positive urine cultures (26.6%). This is probably associated with the mechanism by which *E. coli *causes meningitis, as the most common source of infection is UTI [73].

An MRI scan of the brain with intravenous contrast is the most accurate imaging technique for diagnosing a cerebral abscess [77,78]. On MRI, a brain abscess typically appears as a ring-enhancing lesion with a hypointense necrotic center and an iso- or hyperintense capsule on T1-weighted images [77]. The necrotic center of the abscess exhibits a hyperintense signal, whereas the capsule appears as hypointense on T2-weighted images [78,81]. A CT scan typically reveals a lesion with a hypodense core encircled by a thin, contrast-enhancing capsule, and sometimes accompanied by perifocal edema [77,78]. Factors such as the abscess location, number, diameter, and any induced midline shift are important radiological indicators for prognosis [82]. Since 2000, brain abscesses have been reported in only eight patients [17,19-21,27,37]. On the other hand, pneumocephalus (air/gas in the intracranial spaces) is a rare and serious complication of bacterial meningitis, usually diagnosed using a brain CT scan (air bubble sign) [83]. In this review, pneumocephalus was observed in four cases and was associated with an increased likelihood of mortality during hospitalization [24,25,30,41]. Other frequent complications in patients with *E. coli* meningitis include the presence of new cerebral infarctions (7.9%), organ failure (7.9%), hydrocephalus (3.1%), and septic shock (3.9%). The thrombotic effect of *E. coli *meningitis may be associated with endotoxemia or systemic inflammation [84].

Septicemia, septic shock, organ failure, and inappropriate antibiotic treatment may be associated with poor outcomes [17,22]. In this study, bacteremia, organ failure, immediate antibiotic use, and the presence of ESBL *E. coli *were not associated with mortality. Empirical antibiotic therapy, typically comprising a third-generation cephalosporin and metronidazole for a minimum period of six weeks, is suggested [78]. However, in case of a brain abscess, the recommended minimum duration of intravenous antibiotic therapy ranges from four to 12 weeks [81]. Early neurosurgical intervention is generally recommended for abscesses larger than 25 mm or in cases where an elevated intracranial pressure is suspected by the clinical manifestations [82,85]. Initially, our patient was managed conservatively with a regimen of intravenous ceftriaxone, vancomycin, ampicillin, and acyclovir, tailored to his clinical presentation, immunodeficiency, and past infectious history. This regimen was subsequently adjusted to ceftriaxone and metronidazole based on the confirmation of *E. coli *in the CSF and blood cultures. However, due to clinical deterioration and radiological evidence of abscess enlargement, a decision was made to refer him for neurosurgical craniotomy and abscess drainage. Among 49 patients with *E. coli *meningitis, 90.7% and 73.3% received third or fourth generation cephalosporines as an empirical and final treatment, respectively, followed by penicillin derivatives [10,14,17,27-29,31,33,35,40,42-46,48-50,52-56]. The most common resistance to antibiotics was to ampicillin (10.8%), followed by cephalosporines [10,11,20,22-24,26-29,31,33,36,39,40,42,44,45,48,52-56]. Surgical treatment was reported in eight cases with *E. coli *meningoencephalitis [24,31,34,43,44,47,50], while half of the patients required ICU admission during hospitalization [10,11,14,18,22-31,33-56].

There are certain limitations of our study. Firstly, most of the data were recorded retrospectively from case reports with limited and missing information, as not all authors provided adequate data. Moreover, the number of patients was too small to make significant correlations. Another limitation is the variation in the units of the laboratory examinations across different cases. Finally, there is insufficient information regarding the exact time of symptom onset, imaging studies, and the initiation of the appropriate therapy. As a result, this case report and review serve an informative purpose, and no definitive conclusions can be drawn. Further observational studies are needed to shed light on the risk factors associated with high mortality in *E. coli* meningitis.

## Conclusions

Community-acquired *E. coli *meningoencephalitis in adults, although rare, carries a high mortality rate (possibly near 40%), and conditions such as immunosuppression, *E. coli *urinary tract infections, and diabetes may be key predisposing factors. Fever, altered mental status, and neck stiffness appear to be the most consistent presenting features, while laboratory markers (e.g., elevated CRP levels, very low CSF glucose) and imaging findings, such as pneumocephalus, could identify patients at greatest risk. These observations suggest that early collection of blood and CSF cultures, empiric third-generation cephalosporin therapy with rapid escalation to carbapenem if ESBL production is suspected, and prompt neurosurgical consultation might improve clinical outcomes. Nevertheless, our data have several limitations because they were derived mainly from small, retrospective case series and isolated reports. The findings should therefore be regarded as hypothesis-generating rather than definitive, and larger prospective studies are needed to validate them and refine prognostic assessment in this infection.

## Appendices

Appendix 1

**Table 11:** Susceptibility result for E.coli isolated from cerebrospinal fluid cultures.

Antibiotic tested	Results
Ampicillin	Susceptible
Amoxicillin/clavulanic acid	Susceptible
Amikacin	Susceptible
Aztreonam	Susceptible
Ceftazidime	Susceptible
Ciprofloxacin	Susceptible
Ceftriaxone	Susceptible
Colistin	Susceptible
Cefotaxime	Susceptible
Cefuroxime	Susceptible
Ertapenem	Susceptible
Cefepime	Susceptible
Fosfomycin	Susceptible
Imipenem	Susceptible
Levofloxacin	Susceptible
Cefoxitin	Susceptible
Meropenem	Susceptible
Cotrimoxazole/trimethoprim	Susceptible
Tetracycline	Susceptible
Tigecycline	Susceptible
Tobramycin	Susceptible
Tazobactam/piperacillin	Susceptible
Gentamicin	Susceptible

Appendix 2

**Table 12:** Median age, gender, background, and clinical presentation of community acquired E. coli meningitis cases from January 2000 to January 2024. AIDS: acquired immune deficiency syndrome; UTI: urinary tract infection; MMF: mycophenolate mofetil; n/a: not available; CKD: chronic kidney disease; COVID-19: coronavirus disease 2019; CSF: cerebrospinal fluid; COPD: chronic obstructive pulmonary disease; CLL: chronic lymphocytic leukemia

References	Number of cases (n)	Median age or age (years)	Gender (male/female, n)	Background/risk factors (disease, n)	Clinical manifestation (symptoms, n)	Neck stiffness (n/a or yes/no)	Classic meningitis triad (fever, neck stiffness, altered mental status) (n/a or yes/no)	Duration of symptoms (hours/days)	GCS (median)	GCS 9-14 (altered mental status, n, yes/no)	GCS <9 (coma, n, yes/no)
Bodilsen et al. [11]	36	69, 50-89	M (n=21), F (n=15)	Immunosuppression solid cancer (n= 5), hematological cancer (n=2), drugs (n=7), AIDS (n=1), UTI *E. coli* (n=9), other pathogens (n=13), pneumonia (n=3), septic arthritis (n=2), psoas abscess (n=1), pancreatitis (n=1), ear infection (n=1), alcohol abuse (n=5), liver disease (n=2), diabetes mellitus (n=3), chronic CSF leakage (n=1)	Altered mental status (n=20), seizures (n=2), headache (n=16), fever (n=14), nausea/vomiting (n=13)	23	8	<24 h (n=20)	13	20	9
van Veen et al. [14]	1	67	n/a	Kidney transplantation- immunosuppressive drugs (MMF, cyclosporine, prednisone)	Confusion, nausea, diarrhea	1	-	5d	n/a	n/a	n/a
Hsiao et al. [17]	12	60.5, 33-78	M (n=6), F (n=6)	Nasopharyngeal carcinoma (n=1), myelodysplastic syndrome (n=1), non-Hodgkin lymphoma (n=1), multiple sclerosis (n=1), substance abuse (n=1), alcohol abuse (n=1), liver cirrhosis (n=1), diabetes mellitus (n=5), old cerebral infarct (n=2), hypertension (n=1)	Altered mental status (n=3), seizures (n=2), headache (n=2), fever (n=5), septic shock (n=4)	-	-	n/a	n/a	n/a	n/a
Yang et al. [20]	6	57, 48-66	M (n=4), F (n=2)	Myelodysplastic syndrome (n=1), UTI (not *E. coli*, n=1), CKD on hemodialysis (n=1), alcohol abuse (n=1), diabetes mellitus (n=3), old cerebral infarct (n=2)	Altered mental status (n=7), seizures (n=2), headache (n=1), septic shock (n=3)	-	-	n/a	n/a	n/a	n/a
Rau et al. [19]	3	n/a	n/a	Diabetes mellitus (n=1), healthy (n=1)	n/a	n/a	n/a	n/a	n/a	n/a	n/a
Huang et al. [18]	4	n/a	n/a	Alcohol abuse (n=1), diabetes mellitus (n=3), healthy (n=1)	n/a	n/a	n/a	n/a	n/a	n/a	n/a
Moussiegt et al. [22]	29	66, 58-76	M (n=18), F (n=11)	Immunosuppression (n=6), UTI (*E. coli*, n=14), alcohol abuse (n=4), diabetes mellitus (n=1), concomitant strongyloidiasis (n=3), distant focus of infection (n=20)	Headache (n=9), fever (n=23), nausea and vomiting (n=10)	14	2	n/a	12	n/a	n/a
Lu et al. [21]	4	59.5, 55-66	M (n=2), F (n=2)	Myelodysplastic syndrome (n=1), alcohol abuse (n=1), diabetes mellitus (n=3), old cerebral infarct (n=2), endocarditis (n=1)	Altered mental status (n=2), seizures (n=2), headache (n=1), fever (n=3), septic shock (n=2)	-	n/a	n/a	n/a	n/a	n/a
Bichon et al. [10]	2	50.5 (34-67)	M (n=1), F (n=1)	Sciatica-immunosuppressive drug (corticosteroids n=1), chronic otitis (n=1), alcohol abuse (n=1)	Altered mental status (n=2), seizures (n=1), fever (n=1), septic shock (n=1)	1	1	n/a	8	1	1
Komsani et al. [45]	1	72	F	Gout-immunosuppressive drug (corticosteroids), non-dialysis dependent CKD, nephrolithiasis, multiple UTI in past, cardiomyopathy, atrial fibrillation, anemia, or chronic disease	Altered mental status, weakness, headache	Yes	No	5 days	14	Yes	No
Mir et al. [48]	1	47	M	Diabetes mellitus, hypertension, distant focus of infection (gastroenteritis/food poisoning)	Altered mental status, fever, chills, headache, weakness, diarrhea	Yes	Yes	<24 h	8	No	Yes
Ray et al. [51]	1	n/a, middle-aged	F	n/a	n/a	n/a	n/a	n/a	n/a	n/a	n/a
Memia et al. [46]	1	63	M	Distant focus of infection (UTI by *E. coli*), alcohol abuse, diabetes mellitus, hypertension, hyperlipidemia	Altered mental status	No	No	n/a	10	Yes	No
Amulele et al. [26]	1	n/a, middle-aged	M	Alcohol abuse, smoking	Fever, headache, confusion, diarrhea, vomiting	No	No	7 days	n/a	n/a	n/a
Jeter et al. [37]	1	37	M	Hepatic steatosis, cholelithiasis, smoking	Fever, headache, weakness, diarrhea, vomiting	No	No	7 days	15	No	No
Hurn et al. [35]	1	70	F	Hypertension, COVID-19	Altered mental status, fever, chills, weakness, headache, hearing impairment	No	No	1 day	n/a	Yes	No
Hare et al. [34]	1	60	M	Ankylosing spondylitis-immunosuppressive drug (etanercept)	Altered mental status	No	No	n/a	n/a	n/a	n/a
Dhir et al. [30]	1	36	F	n/a	Altered mental status, fever, vomiting, abdominal pain	Yes	Yes	7 days	13	Yes	No
Zafar et al. [56]	1	35	M	Hypertension	Fever, altered mental status, headache, vomiting, seizures	Yes	Yes	n/a	8	Yes	No
Vale et al. [54]	1	73	F	Lupus erythematosus-immunosuppressive drug (corticosteroids, azathioprine)	Fever, altered mental status	Yes	Yes	2 days	13	Yes	No
Algaba et al. [27]	1	58	M	Distant focus of infection (UTI by *E. coli*)	Fever, altered mental status, dysuria, frequent urination, headache, vomiting, septic shock	No	No	n/a	n/a	n/a	n/a
Kasimahanti et al. [40]	1	56	Μ	CSF leak (rhinorrhea) and repair 23 years prior	Fever, altered mental status, headache, vomiting	No	No	2 days	9	Yes	No
Khanna et al. [42]	1	54	M	n/a	Altered mental status, headache, seizures, tachypnea	No	No	<24 h	11	Yes	No
Tosa et al. [53]	1	78	M	Distant focus of infection (otitis media), cholesteatoma, smoking, atrial fibrillation	Fever, altered mental status, urinary incontinence	Yes	Yes	<24 h	8	No	Yes
Dahal et al. [29]	1	59	F	Rheumatoid arthritis-immunosuppressive drug (corticosteroid, methotrexate, abatacept), diabetes mellitus, distant focus of infection (strongyloidiasis)	Fever, altered mental status	No	No	7 days	n/a	n/a	n/a
Ishida et al. [36]	1	64	Μ	Distant focus of infection (UTI by *E. coli*)	Fever, altered mental status, seizures	No	No	<24 h	9	Yes	No
Kohlmann et al. [44]	1	53	F	Smoking, COPD, vertebral disk herniation, obesity	Altered mental status, fever, neck pain, flu-like symptoms, intermittent diarrhea	Yes	Yes	n/a	n/a	n/a	n/a
Kobayashi et al. [43]	1	78	F	Distant focus of infection (UTI by *E. coli*), spinal stenosis	Altered mental status, back pain	No	No	n/a	n/a	n/a	n/a
Kenzaka and Ueda [41]	1	82	F	Distant focus of infection, UTI by *E. coli*, endocarditis, hypertension	Fever, altered mental status, dysuria	Yes	Yes	3 days	6	No	Yes
Abhilash and Gigin [23]	1	65	M	Distant focus of infection (UTI by *E. coli*), alcohol abuse, diabetes mellitus, hepatic steatosis, hypertension, smoking	Altered mental status, fever, abdominal pain, partial ptosis on left side	Yes	Yes	n/a	7	No	Yes
Kangath and Midturi [39]	1	48	F	Distant focus of infection (UTI by *E. coli*), Marfan syndrome	Fever, headache, left-sided back pain	No	No	n/a	15	No	No
Elaldi et al. [31]	1	84	M	Chronic otitis media	Fever, altered mental status, headache, vomiting, ptosis of left eyelid	Yes	Yes	2 days	n/a	n/a	n/a
Gon et al. [33]	1	72	F	Diabetes mellitus	Fever, lower back pain, altered mental status	No	No	5 days		Yes	No
Weyrich et al. [55]	1	59	n/a	Alcohol abuse, smoking	Fever, altered mental status, headache, nausea, septic shock	Yes	Yes	2 days	12	Yes	No
Lasboo et al [25]	1	84	F	Distant focus of infection (spondylodiscitis)	Altered mental status, urinary incontinence, diarrhea, lower back pain, nausea, vomiting, chills	No	No	9 days	n/a	Yes	No
Miletic et al. [47]	1	39	M	Anterior sacral meningocele	Fever, headache, photophobia	No	No	n/a	n/a	n/a	n/a
Adamides et al. [24]	1	91	M	Distant focus of infection (UTI by *E. coli*), diabetes mellitus	Altered mental status, reduced mobility, right-sided hemiparesis	No	No	2 days	12	Yes	No
Sule and Tai [52]	1	78	F	Hypertension, ischemic heart disease, chronic cholecystitis	Fever, altered mental status	No	No	n/a	n/a	Yes	No
Chang et al. [28]	1	72	F	n/a	Fever, altered mental status	Yes	Yes	1 day	n/a	Yes	No
Fujitani et al. [32]	1	n/a	n/a	n/a	Fever, altered mental status	No	n/a	n/a	n/a	n/a	n/a
Passeron et al. [50]	1	43	M	Alcohol abuse, episodes of *E. coli* meningitis in past, Aspergillus sphenoid sinusitis	Fever, headache	No	No	n/a	15	No	No
Johnson et al. [38]	1	72	M	Distant focus of infection (UTI by *E. coli*), alcohol abuse, liver cirrhosis	Altered mental status, nausea, vomiting, diarrhea	No	No	<24 h	n/a	Yes	No
Mofredj et al. [49]	1	53	M	Alcohol abuse	Fall from same height	No	No	<24 h	15	No	No
Present case	1	61	M	CLL, recent corticosteroid treatment for herpetic cheilitis, otitis media, mastoiditis, hypertension, hyperlipidemia	Fever, altered mental status, seizures, earache	Yes	Yes	<24 h	10	Yes	No

Appendix 3

**Table 13:** Laboratory Examinations and imaging studies of community-acquired E. coli meningitis cases from January 2000 to January 2024. CRP: C-reactive protein; WBC: white blood cells; CSF: cerebrospinal fluid; CT: computed tomography; n/a: not available; MRI: magetic resonance imaging; PET-CT: positron emission tomography; US: ultrasound

References	CRP (median or value, mg/L)	WBC (median count, IQR or value/μL)	CSF leucocyte count (median or leucocyte/mm^3^, leucocyte type)	CSF protein count (median or value, mg/dL)	CSF glucose count (median or value in mg/dL)	Blood or urine or stool or other culture positive for *E. coli *(type of culture, n)	CT of the brain (performed: yes/no, findings, n)	Other imaging studies performed (type, findings)
Bodilsen et al. [11]	230	12600, 6300-20900	2527, n/a	340	n/a	Bacteremia (n=26)	Yes (n=31), infarction (n=5), generalized edema (n=2), hydrocephalus (n=1), cerebritis (n=1)	n/a
van Veen et al. [14]	n/a	n/a	12014, n/a	389	14.4	n/a	Yes, no abnormalities	n/a
Hsiao et al. [17]	n/a	n/a	n/a	n/a	n/a	Bacteremia (n=4)	n/a	n/a
Yang et al. [20]	n/a	n/a	n/a	n/a	n/a	n/a	n/a	n/a
Rau et al. [19]	n/a	n/a	n/a	n/a	n/a	n/a	Yes, brain abscess (n=3)	n/a
Huang et al. [18]	n/a	n/a	n/a	n/a	n/a	n/a	n/a	n/a
Moussiegt et al. [22]	240	7600, 6400-12900	2340, 90% neutrophils	360	12.6	Bacteremia (n=22), bacteriuria (n=14)	n/a	n/a
Lu and Chang [21]	n/a	n/a	5310, n/a	374.75	61.75	Bacteremia (n=3)	n/a	n/a
Bichon et al. [10]	330 (n=1)	2000 (n=1), n/a	523, 90% neutrophils (n=2)	368, 300-436 (n=2)	<1.8 (n=2)	Bacteremia (n=1), bacteriuria (n=1)	Yes (n=2)	n/a
Komsani et al. [45]	n/a	11700	180, 94% neutrophils	97	30	Bacteriuria	Yes, no abnormalities	Abdomen CT (renal subcapsular hematoma, bilateral nephrolithiasis)
Mir et al. [48]	n/a	11500	195, 85% neutrophils	348	123	Bacteremia	Yes, no abnormalities	n/a
Ray et al. [51]	n/a	n/a	n/a	n/a	n/a	n/a	n/a	n/a
Memia et al. [46]	n/a	12000	5954, 80% neutrophils	50	83	Bacteremia, bacteriuria	No	Brain MRI: no abnormalities, abdomen/pelvis CT: acute pyelonephritis
Amulele et al. [26]	n/a	n/a	>999, n/a	355	n/a	n/a	No	No
Jeter et al. [37]	n/a	20800	30, 74% neutrophils	55	65	n/a	Yes, no abnormalities	Brain MRI: abscesses, abdomen US: liver abscess
Hurn et al. [35]	92	12500	n/a	n/a	n/a	n/a	Yes, no abnormalities	Thorax CT: pleural effusion, lower zone consolidation, spine MRI: T10-T11 adhesive arachnoiditis
Hare et al. [34]	n/a	n/a	n/a	n/a	n/a	Positive ventricular drainage culture	n/a	Brain MRI: pyogenic ventriculitis
Dhir et al. [30]	n/a	25000	n/a, 100% neutrophils	n/a	n/a	n/a	Yes, pneumocephalus	n/a
Zafar et al. [56]	n/a	15000	379, 84% neutrophils	240	14	n/a	Yes, no abnormalities	n/a
Vale et al. [54]	42	9800	185, 85% neutrophils	423	<5	Bacteremia	Yes, no abnormalities	PET-CT: osteomyelitis, multifocal arthritis, spine MRI: sacroiliitis
Algaba et al. [27]	n/a	n/a	>4000, n/a	380	<2	Bacteremia, bacteriuria	Yes, diffuse edema	Brain MRI: abscesses
Kasimahanti et al. [40]	n/a	14760	360, 96% neutrophils	>300	<20	n/a	Yes, cystic gliosis changes communicating with the frontal horn of ipsilateral lateral ventricle	Brain MRI: ventriculitis, abdomen CT: renal calculus
Khanna et al. [42]	n/a	22900	4257, n/a	1341	1	n/a	Yes, meningeal enhancement	n/a
Tosa et al. [53]	50	11930	656, 64% neutrophils	302	45	Positive ear discharge culture, positive stool culture	Yes, pneumatization mastoid cells and a shadow in the soft tissue from the right epitympanum to the middle ear	Chest, abdomen, pelvis CT: no abnormalities, brain MRI: cerebral atrophy
Dahal et al. [29]	n/a	16600	330, 98% neutrophils	458	<10	Bacteremia, stool culture (Strongyloides)	No	Abdomen CT: colitis
Ishida et al. [36]	135	n/a	732, 84% neutrophils	169	37	Bacteremia, bacteriuria	Yes, no abnormalities	n/a
Kohlmann et al. [44]	164	33500	1657, n/a	n/a	n/a	Bacteremia, bacteriuria, positive retropharyngeal abscess culture	No	Spine MRI: descending retropharyngeal abscess with cervical spondylodiscitis at C2-C3 to C4-C5, spinal canal stenosis
Kobayashi et al. [43]	n/a	n/a	n/a	n/a	n/a	Bacteremia, bacteriuria, positive ventricular drainage culture	Yes, ventriculitis	n/a
Kenzaka and Ueda [41]	n/a	n/a	85000, n/a	50	40	Bacteremia, bacteriuria	Yes, infarctions, pneumocephalus, ventriculitis	Brain and chest MRI: infectious cerebral embolism, discitis, infective endocarditis
Abhilash and Gigin [23]	n/a	17500	1600, 80% neutrophils	n/a	n/a	Bacteremia	No	Abdomen US: pyelonephritis, cystitis, fatty liver, gall bladder calculus
Kangath and Midturi [39]	n/a	n/a	1400, 83% neutrophils	>200	4	Bacteriuria	No	Spine MRI: meningitis and dural ectasia with intrasacral meningoceles extending into the pelvis along with alterations in the structure of the lumbar vertebral bodies. Abdomen and pelvis CT, IV pyelogram: no abnormalities, abdomen US: no abnormalities
Elaldi et al. [31]	201	13700	2200, 90% neutrophils	495	4	Positive ear discharge culture, positive stool culture	Yes, communicating hydrocephalus, CSF leakage, acute purulent meningitis	n/a
Gon et al. [33]	460	7900	287, 90% neutrophils	403	13	Bacteremia, bacteriuria	Yes, choroid plexus cyst formation, ventricular enlargement	Brain MRI: ventriculitis, MRI spine: L4-L5 spondylitis, abdominal CT: pyelonephritis
Weyrich et al. [55]	292	3850	440, 62% neutrophils	1035	0.18	Bacteremia	Yes, no abnormalities	Brain MRI: no abnormalities, abdomen CT, chest: mycotic aneurysms of the infra-renal aorta, aorto-iliac bifurcation and primitive iliac artery, inferior vena cava thrombosis, kidney infarct
Lasboo et al. [25]	n/a	24300	33500	n/a	n/a	Bacteremia, bacteriuria, positive pus aspiration of intervertebral disc	Yes, pneumocephalus	Brain MRI: meningitis, MRI spine: T11-T12, L1-L2 and L5-S1 discitis, chest, abdomen, and pelvis MRI: psoas muscle abscesses, retrocrural abscesses, lung infarcts, hypodense lesions in the liver
Miletic et al. [47]	n/a	n/a	n/a	n/a	n/a	n/a	n/a	Abdominal US: no abnormalities, CT: sacral bone defect, retrorectal expansive mass, spine MRI: anterior sacral meningocele, cord tethering
Adamides et al. [24]	320	13500	n/a	n/a	n/a	Bacteriuria, positive subdural collection	Yes, pneumocephalus, subdural empyema	n/a
Sule and Tai [52]	127	22000	28, 93% neutrophils	23	55.8	Bacteremia	Yes, lacunar infarcts	n/a
Chang et al. [28]	n/a	23800	5500, 96% neutrophils	105	5.04	Bacteremia	No	n/a
Fujitani et al. [32]	n/a	n/a	n/a	n/a	n/a	n/a	n/a	n/a
Passeron et al. [50]	n/a	5100	14, n/a	123	46.8	Bacteremia, bacteriuria	Yes, sphenoidal sinusitis	Spine MRI, chest, and abdominal CT, prostate and cardiac US: no abnormalities. Sinus CT: sphenoidal sinusitis
Johnson et al. [38]	n/a	n/a	9450, 92% neutrophils	n/a	n/a	Bacteremia, bacteriuria	No	Abdominal US and CT: no abnormalities, index finger X-ray: acute osteomyelitis
Mofredj et al. [49]	n/a	12300	1310, 84% neutrophils	610	66.6	n/a	Yes, no abnormalities	Abdominal US: liver enlargement
Present case	14	14000	3200, 80% neutrophils	309	47	Bacteremia, positive brain abscess collection	Yes, cerebritis, brain abscess, mastoiditis	Brain MRI: mastoiditis, brain abscess

Appendix 4

**Table 14:** Complications, distant focus of infection, causes of death, and treatment choices of community-acquired E. coli meningitis cases from January 2000 to January 2024. ICU: intensive care unit; UTI: urinary tract infection; HHS: hyperosmolar hyperglycemic syndrome; n/a: not available; CSF: cerebrospinal fluid; DIC: disseminated intravascular coagulation; ESBL: extended-spectrum beta-lactamase; n/a: not available

References	Complications during hospitalization	Survivors and neurologic sequelae/other complications on discharge	Concomitant distant focus of infection	Cause and median time (days, range) to death	Initial empiric therapy	Treatment duration (days), onset of suitable treatment (hours/days)	Final treatment (medical or/and surgical) n	Resistance to antibiotics, ESBL	ICU admission (yes/no) n
Bodilsen et al. [11]	Cerebral infarct (n=5), generalized brain edema (n=2), hydrocephalus (n=1), cerebritis (n=1)	Neurologic deficits (n=4)	UTI by *E. coli* (n=9), pneumonia (n=3), septic arthritis (n=2), ear infection (n=1), pancreatitis (n=1), psoas abscess (n=1)	4 (1-26), sepsis (n=7), pneumonia (n=3), respiratory failure (n=2)	n/a	n/a	n/a	n/a	Yes (n=17), intubation (n=7)
van Veen et al. [14]	Hearing impairment (n=1)	Hearing impairment (n=1)	n/a	Survived	Ceftriaxone, penicillin (n=1)	n/a	Ceftriaxone (n=1)	n/a	No
Hsiao et al. [17]	Hyponatremia (n=2), hydrocephalus (n=4), brain abscess (n=1), cerebral infarct (n=3)	From slight to severe neurologic disability (n=4)	n/a	n/a	Ceftriaxone/ceftazidime/cefepime, vancomycin (n=25)	n/a	n/a	Ampicillin (n=10), ceftazidime (n=1), ceftriaxone (n=2), cefepime (n=1), ciprofloxacin (n=3)	n/a
Yang et al. [20]	HHS (n=1), brain abscess (n=1)	n/a	n/a	n/a, 6 (4-8)	n/a	16.8 (mean), n/a (n=6)	n/a	Ceftazidime (n=1), ceftriaxone (n=2)	n/a
Rau et al. [19]	Brain abscess (n=3)	n/a	n/a	n/a	n/a	n/a	n/a	n/a	n/a
Huang et al. [18]	n/a	n/a	n/a	Survived	n/a	n/a	n/a	n/a	No
Moussiegt et al. [22]	Any organ failure (n=10), neurological complication (n=14)	n/a	UTI by *E. coli* (n=14), Strongyloides infestation (n=3)	n/a	n/a	21 (median), n/a (n=24)	3rd generation cephalosporine (n=24)	Ciprofloxacin (n=4), ESBL (n=2)	Yes (n=24)
Lu and Chang [21]	HHS (n=1), septic shock (n=2), brain abscess (n=1)	Hemiparesis (n=1), dysphagia (n=1)	Chronic otitis media (n=1)	n/a	n/a	n/a	n/a	n/a	n/a
Bichon et al. [10]	n/a	Anosognosia (n=1)	UTI (n=1), chronic otitis (n=1)	1, sepsis (n=1)	Ceftriaxone, amoxicillin, acyclovir (n=2)	21 (n=1), <24 h (n=2)	Ceftriaxone (n=2), amoxicillin (n=1)	Trimethoprim-sulfamethoxazole (n=1)	Yes, intubation (n=1)
Komsani et al. [45]	n/a	n/a	UTI by *E. coli* and *Klebsiella pneumoniae* (n=1)	Survived	Ceftriaxone, ampicillin, acyclovir (n=1)	21, <24 h (n=1)	Ceftriaxone (n=1)	n/a	No (n=1)
Mir et al. [48]	Hyponatremia (n=1)	n/a	Gastrointestinal infection (n=1)	Survived	Ceftriaxone, ampicillin, vancomycin, acyclovir (n=1)	21, <24 h (n=1)	Ceftriaxone (n=1)	n/a	No (n=1)
Ray et al. [51]	n/a	n/a	n/a	Survived	n/a	n/a	n/a	n/a	No (n=1)
Memia et al. [46]	n/a	n/a	UTI by *E. coli* (n=1)	Survived	Cefepime, metronidazole (n=1)	21, <24 h (n=1)	Ceftriaxone, ciprofloxacin (n=1)	n/a	No (n=1)
Amulele et al. [26]	n/a	n/a	Gastrointestinal infection (n=1)	Survived	n/a	21, n/a (n=1)	Ceftriaxone (n=1)	Intermediate sensitivity to amoxicillin and amoxicillin-clavulanate (n=1)	No (n=1)
Jeter et al. [37]	Hyponatremia, brain abscesses, liver abscess, hemiparesis (n=1)	n/a	Liver abscess (n=1)	Survived	Piperacillin/tazobactam, micafungin (n=1)	30, n/a (n=1)	Ceftriaxone, metronidazole (n=1)	n/a	No (n=1)
Hurn et al. [35]	Nosocomial pneumonia and UTI (n=1)	Hearing and lower limb motor impairment (n=1)	n/a	Survived	Ceftriaxone, amoxicillin (n=1)	28, <24 h (n=1)	n/a	n/a	Yes, intubation (n=1)
Hare et al. [34]	Pyogenic ventriculitis (n=1)	n/a	n/a	Survived	n/a	n/a	Ceftriaxone, external ventricular drainage (n=1)	n/a	No (n=1)
Dhir et al. [30]	Pneumocephalus (n=1)	Died	n=1	n/a	Meropenem, amikacin, vancomycin (n=1)	3, <24 h (n=1)	Meropenem, amikacin, vancomycin (n=1)	n/a	Yes, intubation (n=1)
Zafar et al. [56]	n/a	n/a	n=1	Sepsis, n/a (n=1)	Ceftriaxone, vancomycin (n=1)	n/a	Meropenem, colistin (n=1)	Ceftriaxone (n=1)	No (n=1)
Vale et al. [54]	Osteomyelitis, multifocal arthritis (n=1)	n/a	Spondylodiscitis (n=1)	Survived	Ceftriaxone, ampicillin, acyclovir (n=1)	56, <24 h (n=1)	Ceftriaxone (n=1)	Amoxicillin, amoxicillin-clavulanate, trimethoprim-sulfamethoxazole (n=1)	No (n=1)
Algaba et al. [27]	Brain abscesses, septic shock (n=1)	n/a	UTI by *E. coli* (n=1)	Survived	Ceftriaxone, ampicillin (n=1)	56, 2 days (n=1)	Ampicillin, ceftriaxone (n=1)	Trimethoprim-sulfamethoxazole (n=1)	Yes, intubation (n=1)
Kasimahanti et al. [40]	n/a	n/a	n=1	Survived	Ceftriaxone, amoxicillin, acyclovir (n=1)	42, 3 days (n=1)	Ceftriaxone (n=1)	Ampicillin (n=1)	No (n=1)
Khanna et al. [42]	n/a	n/a	n=1	Survived	Ceftriaxone, vancomycin (n=1)	n/a, <24 h (n=1)	Ceftriaxone (n=1)	n/a	Yes (n=1)
Tosa et al. [53]	Mastoiditis (n=1)	n/a	Acute otitis media	Survived	Ceftriaxone, ampicillin, vancomycin (n=1)	34, 4 days (n=1)	Meropenem, amikacin (n=1)	Ampicillin, ceftazidime, ceftriaxone, ESBL (n=1)	No (n=1)
Dahal et al. [29]	n/a	Died	Gastrointestinal infection (strongyloidiasis) (n=1)	n/a, 29 (n=1)	Cefepime, ampicillin, ivermectin, albendazole (n=1)	22, <24 h (n=1)	Cefepime, ivermectin, albendazole (n=1)	Piperacillin-tazobactam, cefazolin (n=1)	Yes, intubation (n=1)
Ishida et al. [36]	Septic shock, DIC (n=1)	n/a	UTI by *E. coli* (n=1)	Survived	Ampicillin-sulbactam (n=1)	21, <24 h (n=1)	Cefotaxime, meropenem (n=1)	n/a	Yes, intubation (n=1)
Kohlmann et al. [44]	Nosocomial pneumonia, *Enterococcus faecium *bacteremia, decubitus ulcers, candidemia (n=1)	n/a	Retropharyngeal abscess, spondylodiscitis (n=1)	Survived	Ceftriaxone, ampicillin (n=1)	21, 3 days (n=1)	Meropenem, abscess drainage, and ventral discectomy (n=1)	Cefepime (n=1), ESBL	Yes, intubation (n=1)
Kobayashi et al. [43]	Pyogenic ventriculitis (n=1)	n/a	UTI by *E. coli* (n=1)	Survived	Ceftriaxone (n=1)	n/a	Ceftriaxone, emergent bilateral drainage (n=1)	n/a	No (n=1)
Kenzaka and Ueda [41]	HHS, endocarditis, infectious cerebral embolism, discitis (n=1)	n/a	UTI by *E. coli*, infective endocarditis, spondylodiscitis (n=1)	Survived	n/a	n/a	n/a	n/a	No (n=1)
Abhilash and Gigin [23]	n/a	n/a	UTI (n=1)	Survived	Meropenem (n=1)	14, 3 days (n=1)	Imipenem (n=1)	Meropenem (n=1)	No (n=1)
Kangath and Midturi [39]	n/a	n/a	UTI by *E. coli* (n=1)	Survived	n/a	28 (n=1)	Ceftriaxone (n=1)	n/a	No (n=1)
Elaldi et al. [31]	Hydrocephalus, CSF leakage, mastoiditis (n=1)	n/a	Chronic otitis media (n=1)	Survived	Ceftriaxone (n=1)	30, 2 days (n=1)	Meropenem, amikacin (IV, intraventricular), mastoidectomy, external ventricular drainage (n=1)	Ampicillin, ceftazidime, cefepime, ciprofloxacin (n=1), ESBL	Yes (n=1)
Gon et al. [33]	Spondylitis, DIC (n=1)	n/a	UTI by *E. coli* (n=1)	Survived	Ceftriaxone, meropenem (n=1)	n/a	Meropenem, gentamicin, vancomycin (n=1)	n/a (n=1), ESBL	No (n=1)
Weyrich et al. [55]	Septic shock, mycotic aneurysms of the infra-renal aorta, aorto-iliac bifurcation, and primitive iliac artery, inferior vena cava thrombosis, kidney infarct (n=1)	Died	n/a	31, hemorrhagic shock (n=1)	Cefotaxime, amoxicillin, gentamycin (n=1)	21, n/a (n=1)	Meropenem, ciprofloxacin (n=1)	Ceftazidime, ceftriaxone, cefepime (n=1), ESBL	Yes, intubation (n=1)
Lasboo et al. [25]	Solid organ abscesses, pneumocephalus (n=1)	Died	UTI by *E. coli*, spondylodiscitis, psoas muscle abscess, retrocrural abscess (n=1)	13, sepsis (n=1)	n/a	13, n/a (n=1)	n/a	n/a	No (n=1)
Miletic et al. [47]	Fecal and urinary incontinence, saddle anesthesia (n=1)	n/a	n/a	n/a	n/a	n/a	Posterior laminectomy and dural opening, obliteration of communication between meningocele and intrathecal compartment, CT-guided percutaneous drainage through the ischiorectal fossa	n/a	No (n=1)
Adamides et al. [24]	Pneumocephalus, subdural empyema, peri-operative myocardial infarction, multiple focal seizures (n=1)	Died	UTI by *E. coli* (n=1)	n/a	n/a	n/a	Ceftriaxone, metronidazole, fronto-parietal craniotomy, and drainage (n=1)	n/a (n=1)	No (n=1)
Sule and Tai [52]	Non-STEMI, bilateral lacunar infarcts (n=1)	n/a	Chronic cholecystitis (n=1)	Survived	Ceftriaxone (n=1)	14, <24 h (n=1)	Ceftriaxone (n=1)	n/a (n=1)	No (n=1)
Chang et al. [28]	Hemophagocytic lymphohistiocytosis and hypovolemic shock (n=1)	Died	n=1	55, hypovolemic shock (n=1)	Penicillin-G, ceftriaxone (n=1)	40, <24 h (n=1)	Penicillin-G, ceftriaxone (n=1)	n/a (n=1)	No (n=1)
Fujitani et al. [32]	n/a	n/a	n/a	n/a	n/a	17, n/a (n=1)	n/a	n/a	n/a
Passeron et al. [50]	Sphenoidal sinusitis	n/a	Aspergillar sphenoidal sinusitis, UTI by *E. coli* (n=1)	Survived	Cefotaxime (n=1)	37, <24 h (n=1)	Amoxicillin, ciprofloxacin, sphenoidotomy (n=1)	n/a	No (n=1)
Johnson et al. [38]	Acute renal injury, acute osteomyelitis of index (n=1)	Died	UTI by *E. coli*, acute osteomyelitis of index finger (n=1)	28, systematic complication (n=1)	Piperacillin-tazobactam (n=1)	28, 3 days (n=1)	n/a	n/a	No (n=1)
Mofredj et al. [49]	n/a	n/a	n=1	Survived	Cefotaxime (n=1)	15, <24 h (n=1)	Amoxicillin (n=1)	n/a	No (n=1)
Present case	Brain abscess, mastoiditis	n/a	Acute media otitis, mastoiditis (n=1)	Survived	Ceftriaxone, ampicillin, acyclovir, vancomycin (n=1)	56, <24 h (n=1)	Ceftriaxone, metronidazole, abscess drainage (n=1)	n/a (n=1)	Yes (n=1)

Appendix 5

**Table 15:** Correlation of gender, clinical manifestations, treatment choices, and antibiotic resistance with survival. ICU: intensive care unit; ESBL: extended spectral beta lactamase

Variables	Survivors with *E. coli* meningitis							
	Gender (male)	Headache	Septic shock	Bacteremia	New neurological deficits	ICU admission	Administration of suitable treatment in <24h	ESBL *E. coli*
Correlation coefficient	0.165	0.272	-0.204	-0.232	0.122	-0.226	-0.073	0.035
p-Value	0.335	0.102	0.221	0.182	0.466	0.176	0.726	0.869

**Table 16:** Correlation of risk factors with survival. RF: risk factor; CSF: cerebrospinal fluid

Variables	Survivors with *E. coli *meningitis											
	Present RF	RF immunosuppression	RF chronic otitis	RF sinusitis	RF distant focus of infection	RF chronic CSF leakage	RF alcohol abuse	RF liver cirrhosis	RF liver disease	RF diabetes mellitus	RF hypertension	RF smoking
Correlation coefficient	0.371	-0.075	0.130	0.588	-0.162	0.090	-0.196	-0.316	0.130	-0.120	0.286	0.060
p-Value	0.079	0.653	0.437	0.090	0.331	0.588	0.239	0.058	0.437	0.473	0.086	0.720

Appendix 6

**Table 17:** Correlation of CT findings/complications with survival. CT: computed tomography

Variables	Survivors with *E. coli *meningitis			
	CT abnormalities	Cerebral infarct	Brain abscess	Pneumocephalus
Correlation coefficient	0.073	0.149	0.186	-0.436
p-Value	0.681	0.399	0.294	0.014

Appendix 7

**Table 18:** Regression analysis to describe the correlation between C-reactive protein (CRP) and cerebrospinal fluid (CSF) levels, as well as the presence of pneumocephalus, and survival.

Survivors with *E. coli* meningitis			
Variables	Regression analysis		
	OR	95% CI	p-Value
CRP	0.988	0.975, 1.002	0.106
CSF glucose levels	1.344	0.766, 2.359	0.303
Pneumocephalus	0.071	0.005, 0.942	0.045

## References

[1] Chang CJ, Chang WN, Huang LT (2004). Bacterial meningitis in infants: the epidemiology, clinical features, and prognostic factors. Brain Dev.

[2] Hallmaier-Wacker LK, Andrews A, Nsonwu O, Demirjian A, Hope RJ, Lamagni T, Collin SM (2022). Incidence and aetiology of infant Gram-negative bacteraemia and meningitis: systematic review and meta-analysis. Arch Dis Child.

[3] Taziarova M, Holeckova K, Lesnakova A (2007). Gram-negative bacillary community acquired meningitis is not a rare entity in last two decades. Neuro Endocrinol Lett.

[4] Pomar V, Benito N, López-Contreras J, Coll P, Gurguí M, Domingo P (2013). Spontaneous gram-negative bacillary meningitis in adult patients: characteristics and outcome. BMC Infect Dis.

[5] Durand ML, Calderwood SB, Weber DJ, Miller SI, Southwick FS, Caviness VS Jr, Swartz MN (1993). Acute bacterial meningitis in adults. A review of 493 episodes. N Engl J Med.

[6] Oordt-Speets AM, Bolijn R, van Hoorn RC, Bhavsar A, Kyaw MH (2018). Global etiology of bacterial meningitis: a systematic review and meta-analysis. PLoS One.

[7] Harder E, Møller K, Skinhøj P (1999). Enterobacteriaceae meningitis in adults: a review of 20 consecutive cases 1977-97. Scand J Infect Dis.

[8] Lu CH, Chang WN, Chuang YC, Chang HW (1998). The prognostic factors of adult Gram-negative bacillary meningitis. J Hosp Infect.

[9] Dzupova O, Rozsypal H, Prochazka B, Benes J (2009). Acute bacterial meningitis in adults: predictors of outcome. Scand J Infect Dis.

[10] Bichon A, Aubry C, Dubourg G, Drouet H, Lagier JC, Raoult D, Parola P (2018). Escherichia coli spontaneous community-acquired meningitis in adults: a case report and literature review. Int J Infect Dis.

[11] Bodilsen J, Brouwer MC, Kjærgaard N, Sirks MJ, van der Ende A, Nielsen H, van de Beek D (2018). Community-acquired meningitis in adults caused by Escherichia coli in Denmark and the Netherlands. J Infect.

[12] Cabellos C, Verdaguer R, Olmo M (2009). Community-acquired bacterial meningitis in elderly patients: experience over 30 years. Medicine (Baltimore).

[13] van de Beek D, de Gans J, Spanjaard L, Weisfelt M, Reitsma JB, Vermeulen M (2004). Clinical features and prognostic factors in adults with bacterial meningitis. N Engl J Med.

[14] van Veen KE, Brouwer MC, van der Ende A, van de Beek D (2016). Bacterial meningitis in solid organ transplant recipients: a population-based prospective study. Transpl Infect Dis.

[15] Bouadma L, Schortgen F, Thomas R, Wutke S, Lellouche F, Régnier B, Wolff M (2006). Adults with spontaneous aerobic Gram-negative bacillary meningitis admitted to the intensive care unit. Clin Microbiol Infect.

[16] Chen SF, Chang WN, Lu CH (2005). Adult Acinetobacter meningitis and its comparison with non-Acinetobacter Gram-negative bacterial meningitis. Acta Neurol Taiwan.

[17] Hsiao WC, Lee JJ, Chien CC, Lu CH, Chang WN, Lien CY (2021). The clinical characteristics and therapeutic outcomes of Escherichia coli meningitis in adults. Acta Neurol Taiwanica.

[18] Huang CR, Lu CH, Chang HW, Lee PY, Lin MW, Chang WN (2002). Community-acquired spontaneous bacterial meningitis in adult diabetic patients: an analysis of clinical characteristics and prognostic factors. Infection.

[19] Rau CS, Chang WN, Lin YC (2002). Brain abscess caused by aerobic Gram-negative bacilli: clinical features and therapeutic outcomes. Clin Neurol Neurosurg.

[20] Yang TM, Lu CH, Huang CR (2005). Clinical characteristics of adult Escherichia coli meningitis. Jpn J Infect Dis.

[21] Lu CH, Chang WN (2000). Escherichia coli meningitis in adults: report of 14 cases. Infect Dis Clin Pract.

[22] Moussiegt A, Birgy A, Cointe A, Duval X, Bidet P, Bonacorsi S (2022). Escherichia coli community-acquired meningitis in adults: a case series of 29 patients in France. Clin Microbiol Infect.

[23] Abhilash K, Gigin SV (2013). A rare manifestation of Escherichia coli septicemia. Int J Res Med Sci.

[24] Adamides AA, Goldschlager T, Tulloch SJ, McMahon JH (2007). Pneumocephalus from gas-forming Escherichia coli subdural empyema. Br J Neurosurg.

[25] Lasboo AA, Walker MT, Hijaz TA (2010). An unusual appearance of discitis due to gas-forming Escherichia coli with associated pneumocephalus. Spine (Phila Pa 1976).

[26] Amulele AV, Ong'ayo G, Arara AM (2023). Recurrent spontaneous Escherichia coli meningitis in an adult: a case report. JAC Antimicrob Resist.

[27] Algaba RA, Álvarez-Marín R, Praena J, Smani Y (2019). Escherichia coli causing meningitis in an adult: a case report and experimental characterization of its virulence. Enferm Infecc Microbiol Clin (Engl Ed).

[28] Chang KH, Lyu RK, Tang LM (2006). Spontaneous Escherichia coli meningitis associated with hemophagocytic lymphohistiocytosis. J Formos Med Assoc.

[29] Dahal S, Lederman J, Berman J, Viseroi M, Jesmajian S (2017). A case of bacteremia and meningitis associated with piperacillin-tazobactam nonsusceptible, ceftriaxone susceptible Escherichia coli during strongyloides hyperinfection in an immunocompromised host. Case Rep Infect Dis.

[30] Dhir A, Dahiya S, Bhardwaj N, Gupta M (2020). Spontaneous extensive pneumocephalous as a rare manifestation of Escherichia coli suppurative meningitis in diabetic ketoacidosis. BMJ Case Rep.

[31] Elaldi N, Gozel MG, Kolayli F, Engin A, Celik C, Bakici MZ, Vahaboglu H (2013). Community-acquired CTX-M-15-type ESBL-producing Escherichia coli meningitis: a case report and literature review. J Infect Dev Ctries.

[32] Fujitani S, Kamiya T, Kaufman L (2005). Community-acquired Escherichia coli meningitis in adult. Hawaii Med J.

[33] Gon Y, Otsubo R, Murase S, Park K, Nakazawa K, Hara H (2012). Case of an elderly patient with community acquired bacterial meningitis due to extended spectrum β lactamase producing Escherichia coli. [Article in Japanese]. Rinsho Shinkeigaku.

[34] Hare D, Doyle D, Doherty A (2021). Spontaneous Escherichia coli meningitis and pyogenic ventriculitis in an adult receiving anti-tumour necrosis factor alpha therapy. Ir Med J.

[35] Hurn E, Dickinson L, Abraham JA (2021). Bacterial meningitis and COVID-19: a complex patient journey. BMJ Case Rep.

[36] Ishida K, Noborio M, Nakamura M, Ieki Y, Sogabe T, Sadamitsu D (2016). Spontaneous Escherichia coli bacterial meningitis mimicking heatstroke in an adult. Clin Case Rep.

[37] Jeter K, Dang A, Ly A, Jayasekara D (2022). Spontaneous Escherichia coli meningitis and brain abscess in an immunocompetent adult. Cureus.

[38] Johnson JR, Gajewski A, Lesse AJ, Russo TA (2003). Extraintestinal pathogenic Escherichia coli as a cause of invasive nonurinary infections. J Clin Microbiol.

[39] Kangath RV, Midturi J (2013). An unusual case of E coli meningitis in a patient with Marfan's syndrome. BMJ Case Rep.

[40] Kasimahanti R, Satish SK, Anand M (2018). Community-acquired Escherichia coli meningitis with ventriculitis in an adult - a rare case report. J Intensive Care.

[41] Kenzaka T, Ueda Y (2013). Infectious cerebral embolism. Rev Assoc Med Bras (1992).

[42] Khanna S, Moore LE, Greyson B (2018). Full neurological recovery from Escherichia coli meningitis associated with near-death experience. J Nerv Ment Dis.

[43] Kobayashi H, Suzuki T, Tokuda Y (2013). Pyogenic ventriculitis following urosepsis caused by Escherichia coli. BMJ Case Rep.

[44] Kohlmann R, Nefedev A, Kaase M, Gatermann SG (2015). Community-acquired adult Escherichia coli meningitis leading to diagnosis of unrecognized retropharyngeal abscess and cervical spondylodiscitis: a case report. BMC Infect Dis.

[45] Komsani MR, Almaghlouth NK, Charla S (2024). Escherichia coli meningitis in a 72-year-old woman. R I Med J.

[46] Memia A, Deda X, Broka A, Kawalet M, Berger J (2023). Escherichia coli meningitis in a patient with urinary tract infection: a case report. Cureus.

[47] Miletic D, Poljak I, Eskinja N, Valkovic P, Sestan B, Troselj-Vukic B (2008). Giant anterior sacral meningocele presenting as bacterial meningitis in a previously healthy adult. Orthopedics.

[48] Mir M, Hassan E, Sharaf A (2023). An unusual case of Escherichia Coli meningitis in an immunocompetent adult. Cureus.

[49] Mofredj A, Guerin JM, Leibinger F, Mamoudi R (2000). Spontaneous Escherichia coli meningitis in an adult. Scand J Infect Dis.

[50] Passeron A, Capron L, Grateau G (2004). Recurrent Escherichia coli meningitis associated with aspergillar sphenoidal sinusitis. Scand J Infect Dis.

[51] Ray A, Basu S, Das S, Chandra A (2023). Gram-negative bacillary meningitis in an immunocompetent adult. BMJ Case Rep.

[52] Sule AA, Tai DY (2007). Spontaneous Escherichia coli meningitis in an adult. Crit Care Shock.

[53] Tosa M, Aihara M, Murakami J (2018). Extended-spectrum beta-lactamase-producing Escherichia coli meningitis that developed from otitis media with cholesteatoma. Intern Med.

[54] Vale F, Pinto Junior VL, Casella MI, Poças J (2019). Community-acquired Escherichia coli meningitis and spondylodiscitis in an adult patient with discoid lupus erythematosus. IDCases.

[55] Weyrich P, Ettahar N, Legout L, Meybeck A, Leroy O, Senneville E (2012). First initial community-acquired meningitis due to extended-spectrum beta-lactamase producing Escherichia coli complicated with multiple aortic mycotic aneurysms. Ann Clin Microbiol Antimicrob.

[56] Zafar M, Tauseef A, Asghar MS (2020). Escherichia coli: a rare cause of meningitis in immuno-competent adult. J Community Hosp Intern Med Perspect.

[57] Pota V, Passavanti MB, Coppolino F (2021). Septic shock due to Escherichia coli meningoencephalitis treated with immunoglobulin-M-enriched immunoglobulin preparation as adjuvant therapy: a case report. J Med Case Rep.

[58] Wang P, Gao B, Wang M, Sheng Q, Tu M (2020). Challenges in the nursing care of intracranial carbapenem-resistant Escherichia coli infection after severe traumatic brain injury: a case report. Ann Palliat Med.

[59] Akuzawa N, Osawa T, Totsuka M, Hatori T, Imai K, Kitahara Y, Kurabayashi M (2014). Secondary brain abscess following simple renal cyst infection: a case report. BMC Neurol.

[60] Nishi H, Shibagaki Y, Hatakeyama S (2005). Metastatic intracranial subdural empyema from renal cyst infection in autosomal dominant polycystic kidney disease. Nephrol Dial Transplant.

[61] Redhu R, Shah A, Jadhav M, Goel A (2011). Spontaneous tension pneumocephalus in a patient with subdural empyema. J Clin Neurosci.

[62] Ren Y, You C, Liu X (2017). A prepontine subarachnoid abscess by Escherichia coli. Neurology.

[63] Scarpa A, Di Stadio A, De Luca P (2021). How can we manage Bezold abscess emergency in COVID-19 pandemic? Blind ceftriaxone and metronidazole treatment to avoid infection spread. J Surg Case Rep.

[64] Aquila I, Gratteri S, Sacco MA, Ricci P (2017). A rare case of fatal meningoencephalitis with septic thromboembolism due to otitis media: a forensic case and review of literature. BMJ Case Rep.

[65] Chang WN, Lu CH, Huang CR, Chuang YC (2000). Mixed infection in adult bacterial meningitis. Infection.

[66] (2023). Global, regional, and national burden of meningitis and its aetiologies, 1990-2019: a systematic analysis for the Global Burden of Disease Study 2019. Lancet Neurol.

[67] Falagas ME, Nikou SA, Siempos II (2007). Infections related to coils used for embolization of arteries: review of the published evidence. J Vasc Interv Radiol.

[68] Roos KL, Tyler KL (2016). Acute meningitis and encephalitis. Harrison's Manual of Medicine. Nineteenth Edition.

[69] Zhou YJ, Wu JN, Chen LJ, Zhao HY (2019). Comparison of infection rate with tunneled vs. standard external ventricular drainage: a prospective, randomized controlled trial. Clin Neurol Neurosurg.

[70] Teckie G, Karstaedt A (2015). Spontaneous adult Gram-negative bacillary meningitis in Soweto, South Africa. Int J Infect Dis.

[71] Coureuil M, Lécuyer H, Bourdoulous S, Nassif X (2017). A journey into the brain: insight into how bacterial pathogens cross blood-brain barriers. Nat Rev Microbiol.

[72] Dietzman DE, Fischer GW, Schoenknecht FD (1974). Neonatal Escherichia coli septicemia - bacterial counts in blood. J Pediatr.

[73] Roos KL, Tyler KL (2018). Brain abscess and empyema. Harrison's Principles of Internal Medicine. Twenty-First Edition.

[74] Anniko M, Bernal-Sprekelsen M, Bonkowsky V, Bradley PJ, Iurato S (2010). Otorhinolaryngology, Head and Neck Surgery. Otorhinolaryngol. Head Neck Surg. Eur. Man. Med.

[75] Wu JF, Jin Z, Yang JM, Liu YH, Duan ML (2012). Extracranial and intracranial complications of otitis media: 22-year clinical experience and analysis. Acta Otolaryngol.

[76] Kaplan DM, Gluck O, Kraus M, Slovik Y, Juwad H (2017). Acute bacterial meningitis caused by acute otitis media in adults: a series of 12 patients. Ear Nose Throat J.

[77] Ruiz-Barrera MA, Santamaría-Rodríguez AF, Zorro OF (2022). Brain abscess: a narrative review. Neurol Perspect.

[78] Sonneville R, Ruimy R, Benzonana N (2017). An update on bacterial brain abscess in immunocompetent patients. Clin Microbiol Infect.

[79] Nigrovic LE, Kimia AA, Shah SS, Neuman MI (2012). Relationship between cerebrospinal fluid glucose and serum glucose. N Engl J Med.

[80] Widdrington JD, Bond H, Schwab U (2018). Pyogenic brain abscess and subdural empyema: presentation, management, and factors predicting outcome. Infection.

[81] Thurnher MM (2019). Neuroimaging in bacterial and mycobacterial infections of the brain. Clinical Neuroradiology.

[82] Campioli CC, Almeida NE, O'Horo JC (2021). Bacterial brain abscess: an outline for diagnosis and management. Am J Med.

[83] Schirmer CM, Heilman CB, Bhardwaj A (2010). Pneumocephalus: case illustrations and review. Neurocrit Care.

[84] Carnevale R, Sciarretta S, Valenti V (2020). Low-grade endotoxaemia enhances artery thrombus growth via Toll-like receptor 4: implication for myocardial infarction. Eur Heart J.

[85] Zhou W, Shao X, Jiang X (2018). A clinical report of two cases of cryptogenic brain abscess and a relevant literature review. Front Neurosci.

